# The effects of *Rhodopseudomonas palustris* PSB06 and CGA009 with different agricultural applications on rice growth and rhizosphere bacterial communities

**DOI:** 10.1186/s13568-019-0897-z

**Published:** 2019-10-31

**Authors:** Luyun Luo, Pei Wang, Zhongying Zhai, Pin Su, Xinqiu Tan, Deyong Zhang, Zhuo Zhang, Yong Liu

**Affiliations:** 1 College of Bioscience & Biotechnology, Hunan Agricultural University, Changsha, China; 2 Hunan Plant Protection Institute, Hunan Academy of Agricultural Science, Changsha, China

**Keywords:** Photosynthetic bacteria, PSB06, CGA009, Rhizosphere microorganism, Rice

## Abstract

In recent years, the photosynthetic bacteria have been used widely in agriculture, but the effects of different agricultural applications on crop rhizosphere microorganism and crops are lack. In this study, we provide new insights into the structure and composition of the rice root-associated microbiomes as well as the effect on crop of the *Rhodopseudomonas palustris(R. palustris)* PSB06 and CGA009 at the rice seedling stage with seed immersion and root irrigation. Compare with CK group, the length of stem, the peroxidase (POD), and superoxide dismutase (SOD) activities in PSB06 treatment group was significantly higher, while the length of stem in CGA009 treatment group was significantly higher. The POD and SOD activities in CGA009 treatment groups only were higher slightly than the CK group. In the study, the dominant phyla were *Proteobacteria* (51.95–61.66%), *Bacteroidetes* (5.40–9.39%), *Acidobacteria* (4.50–10.52%), *Actinobacteria* (5.06–8.14%), *Planctomycetes* (2.90–4.48%), *Chloroflexi* (2.23–5.06%) and *Firmicutes* (2.38–7.30%), accounted for 87% bacterial sequences. The principal coordinate analysis (pCoA) and mantel results showed the two application actions of *R. palustris* CGA009 and PSB06 had significant effects on rice rhizosphere bacterial communities (p < 0.05). The PSB06 can significantly promote the rice growth and enhance stress resistance of rice at the seedling stage, while the *R. palustris* CGA009 has no significant effect on rice. Dissimilarity test and canonical correspondence analysis (CCA) results showed that the TN and pH were the key factors affecting rice rhizosphere bacterial community in the seedling stage. This study will provide some guidance advices for the study of the microecological regulation of photosynthetic bacteria on crops.

## Introduction

The different microbial communities above and below the ground reflect that these microbial communities have plant habitat specificity, and are closely related to plant health. Previous studies indicated that the diversity of leaf communities decreased with the increasing of the distance from the soil to different tomato plant habitats (Ottesen et al. 2013), suggesting that soil may be the main source of the phyllosphere microbial populations. Because of its important function in the Earth’s biogeochemical cycles (Fierer et al. 2012; Philippot et al. 2013), soil microorganisms have been used as an early indicators of soil health status in the soil ecosystem (Hernández-Allica et al. 2006). All plant-related habitats contain a high proportion of plant beneficial microorganisms, plant pathogens, and potential human pathogens. And the dynamic changes in these microorganisms may affect sustainable plant production and plant health (Berg et al. 2005; Mendes et al. 2013). In general, plants drive the composition and structure of rhizosphere bacterial communities through root exudates (Bais et al. 2006; Micallef et al. 2009). In turn, rhizosphere microorganisms can promote the overall health of plant species by promoting crop growth and participating root surface defense protection (Berg et al. 2005; Mendes et al. 2013).

The pathogenic bacteria associated with plant diseases often cause great economic losses to agricultural production. The applications of chemical pesticides and biological control agents (BCAs) usually have been recommended to control disease incidence and severity in agriculture (Erwin and Ribeiro 1996). However, the excessive use of chemical pesticides often leads to environmental and food safety problems (Sharma et al. 2012). Nowadays, more and more BCAs, such as *Bacillus* spp*., Pseudomonas* spp*., Trichoderma* spp. etc., being commercialized for various crops as a desirable strategy for controlling plant diseases (Trabelsi and Mhamdi 2013; You et al. 2015; Cha et al. 2016). The application of these BCAs has greatly promoted the improvement of agricultural production.

The applications of BCAs on plant have primarily focused on the rhizosphere, which can be obtained by manipulating the rhizosphere microorganisms (Janvier et al. 2007; Santhanam et al. 2015). Inoculation BCAs can change the composition of resident microbial communities in the rhizosphere, thereby affecting the soil community’s ecological and functional properties (You et al. 2015). The BCAs could introduce beneficial microorganisms resulting in reduce plant pathogen and insect damage (Hadar and Papadopoulou 2012; Mehta et al. 2014), as well as human pathogens (Mootian et al. 2009; Oni et al. 2015). Therefore, the control of plant disease could be more direct and effective by applying BCAs on the rhizosphere.

The *Rhodopseudomonas palustris* (*R. palustris*) PSB06(CCTCC NO:M2012518), a wild-type stains, as a long-time established BCA, was successfully applied to suppress crop disease and promote plant growth. In previous study, we found that the PSB06 had good effect on controlling various rice diseases. The *R. palustris* CGA009 (ATCC BAA-98), was obtained from the American Type Culture Collection (ACTT, USA), a different physiological subspecies of the *R. palustris*, which is mainly used in hydrogen production research internationally at present studies (Piskorska et al. 2013; Lu et al. 2016). In this study, we also explored the effects agent *R. palustris* CGA009 on rice growth and rhizosphere microorganisms. At present, the seed immersion and root irrigation are mainly used to treat crops at the seedling stage in agriculture. In the previous preliminary experiments, we found that seed immersion with *R. palustris* PSB06 also promoted the crop growth. At the same time, the application of seed soaking method prevented diseases in the seed periods, and then reduced the application of BCAs during the crop growth and development. Therefore, root irrigation and seed soaking were used in this experiment. However, the inoculation of exotic species may alter biological processes in the soil by either direct or indirect actions (You et al. 2015). Hence, it is necessary to study the effect of the *R. palustris* PSB06 and CGA009 on soil micro-environment. In this experiment, the effects of root irrigation and seed soaking on rice growth and rhizosphere microorganisms were evaluated at seedling stage.

We hypothesize the inoculation of *R. palustris* PSB06 and CGA009 can change the rhizosphere bacterial community and affect the rice seedling growth with seed immersion and root irrigation. To test this hypothesis, we investigated the impact of the BCA *R. palustris* PSB06 on the indigenous rhizosphere bacterial community using 16S rRNA amplicon sequencing and growth of seedling rice with seed immersion and root irrigation. In addition, we also explore the effect of agent *R. palustris* CGA009 on rhizosphere bacterial community using 16S rRNA amplicon sequencing and growth of seedling rice by carrying out in-depth studies on the soil micro-environment.

## Materials and methods

### Greenhouse experiments

The experiment was performed in Hunan Plant Protection Institute (28°12′29.81″N, 113°5′35.83″E, Changsha, China) from March 5, 2018 to May 25, 2018. Experimental soils were mixed before transplanting the rice seedlings. The hybrid rice variety C LiangYou number 7 was selected as the experimental material. The seeds were soaked with different treatments for 3 h: Control (CK): sterile water; treatment A (BP): the photosynthetic bacteria agent *R. palustris* PSB06 (CCTCC NO:M2012518); treatment B (BC): the photosynthetic bacteria agent *R. palustris* CGA009 (ATCC BAA-98). Root irrigation with PSB06 and CGA009 after 15 days when transplanting the rice seedling: treatment C(GP): root irrigation with the photosynthetic bacteria agent PSB06, treatment D(GC): root irrigation with the photosynthetic bacteria agent *R. palustris* CGA009. After seeds were soaked with different treatments for 3 h, and then transplanted to the flowerpot with mixed field paddy soil and incubated at 26 °C on 16 h/8 h day/night cycle, the humidity was maintained at 72% at the greenhouse. The root length and seedling height of twenty rice plants were measured on the 14th day after application, and rice leaves were collected. The rice seedlings and soil samples were collected at seedling stage (day 40) with six replicates. The soil samples were divided into two parts, some soil samples were used to determine soil physicochemical properties, while the other soil samples were stored at − 80 °C for DNA extraction.

### Physicochemical properties of rice seedling and soils

The length of root and stalk were measured through a ruler. The activities of superoxide dismutase (SOD) and peroxidase (POD) in rice seedling leaves were determined by the NBT photoreduction method (Min et al. 2014). The pH of soil samples was detected in aqueous extract (soil: deionized water = 1:5) using a multiparameter water quality monitoring instrument. The content of total nitrogen (TN) (modified kjeidahi method, HJ/T 707-2014), available kalium (AK) (flame atomic absorption spectrophptprnetry method, GB 9836-1998), available phosphorus (AP) (sodium hydrogen carbonate solution-Mo-Sb anti spectrophotometric method, HJ/T 704-2014), and organic matter (OM) (Method for determination of soil organic matter, GB9834-1988) were measured by Institute of Soil Science, Chinese Academy of Sciences (Nanjing, China).

### PCR amplification and sequencing

Total soil genomic DNA was extracted with the Fast DNA spin kit for soil (MP Biomedicals LLC, USA). The V4 region of bacterial 16S rRNA gene was amplified as Lin et., al described (Lin et al. 2017). A unique 12 bp barcode sequence was used to distinguish different samples. The PCR was performed with a 50 μL mixture as Lin et. al reported (Lin et al. 2017). The PCR products were purified with the E.Z.N.A.^®^ Gel Extraction Kit (Omega Biotek, Norcross, GA, USA), and the final amplicon was quantified using Qubit™ 2 Fluorometer. The DNA samples were sequenced with Hiseq platform at Puwikon, Co., Ltd (Nanjing, China). We used the in-house pipeline (http://mem.rcees.ac.cn:8080) to process the raw 16S rRNA sequence data which consisted of mount of bioinformatics tools. In brief, the raw reads were assembled and assigned to separate samples with the 12 bp barcodes. And the low-quality sequences with the average quality score less than 20 and an ambiguous basis (N) were removed. Forward and reverse sequences were combined by the bioinformatics software FLASH (Magoc and Salzberg 2011). And then the sequences with length < 200 bp were deleted. Chimeras were removed and OTU (operational taxonomic units) table were generated by the UPARSE software at a 97% similarity level (Edgar 2013). The taxonomy annotation was conducted using the RDP Classifier database (Silva database 132 version) to assign the microbial representative sequences (Abarenkov et al. 2010). Then the resampled OTU table was obtained using the OTU table with the lowest sequences for the downstream analysis (Wang et al. 2007). The all raw sequence data have been submitted to NCBI sequence Read Archive (SRA) under the accession number PRJNA554261.

### Statistical analysis

The difference of top 30 genera between two groups was assessed using the STAMP software v2.1.3. The student *t* test was conducted to compare the growth properties, physicochemical properties, and alpha diversity indices between two groups using the software SPSS 21.0 (IBM Co., Armonk, NY, USA). Correlation analysis between physicochemical properties and bacterial populations were also analyzed with the Spearman method using the software SPSS 21.0 (IBM Co., Armonk, NY, USA). The principal coordinate analysis (pCoA) and the dissimilarity tests (MRPP, Adonis, and ANOSIM) were used to demonstrate the bacterial community differences among the five groups (Anderson 2010; Caporaso et al. 2010). Canonical correspondence analysis (CCA) was used to measure the correlation between the bacterial community and different environmental factors. FAPROTAX was used to establish Functional Annotation of Prokaryotic Taxa (Louca et al. 2016).

## Results

### Growth properties and physicochemical properties of different treatment group

**Fig. 1:**
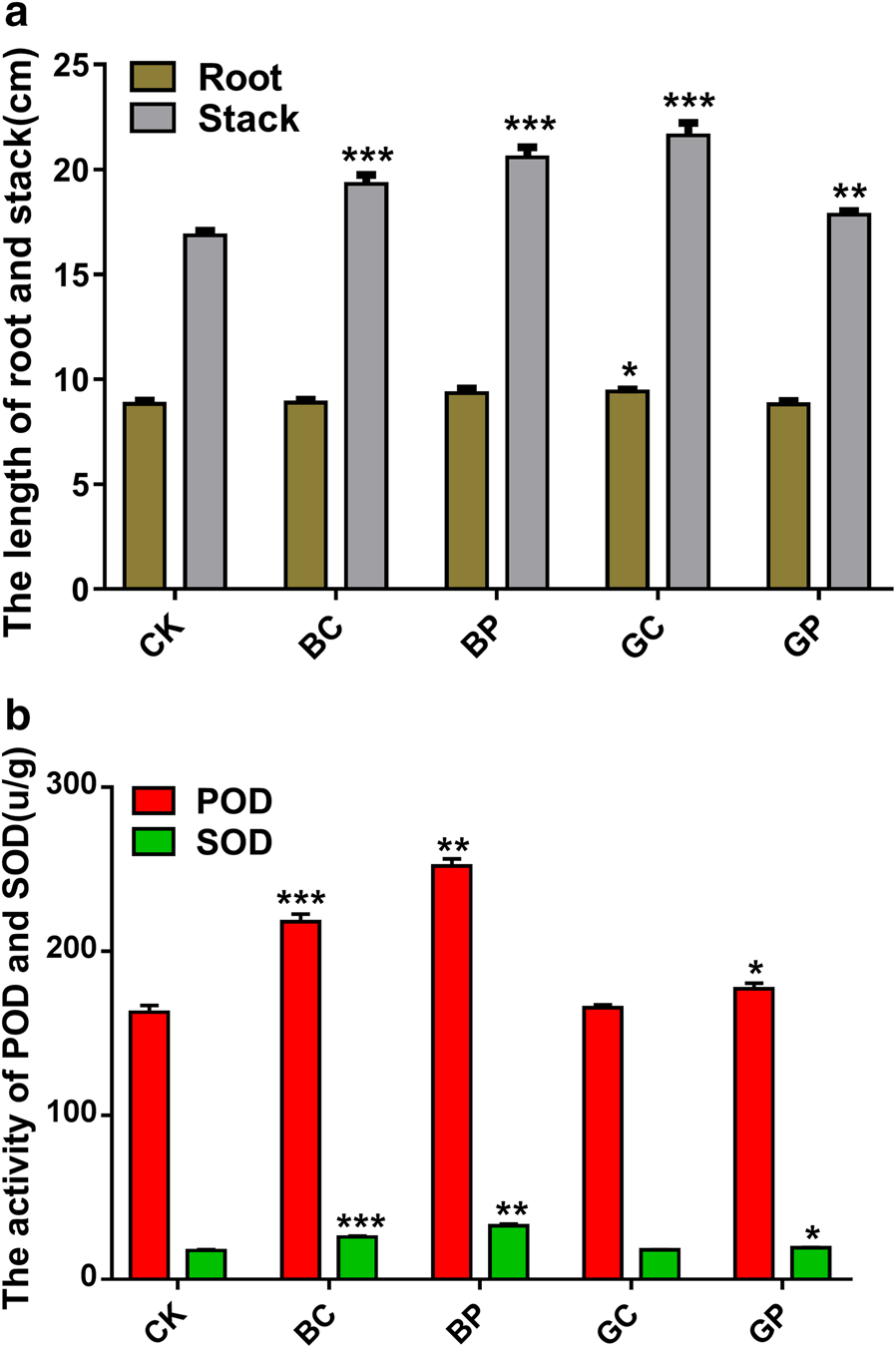
The length of root, stalk (**a**) and the activities of superoxide dismutase (SOD) and peroxidase (POD) in rice seedling leaves (**b**). Control (CK): sterile water; treatment BP: the photosynthetic bacteria agent PSB06; treatment BC: the photosynthetic bacteria agent CGA009; treatment GP: root irrigation with the photosynthetic bacteria agent PSB06; treatment GC: root irrigation with the photosynthetic bacteria agent CGA009

The length of root and stalk were showed in the Fig. 1. The length of root ranges from 8.82 ± 0.15 to 9.42 ± 0.09 cm. The length of pepper stalk in the BC and BP groups, were 19.30 ± 0.44 and 20.57 ± 0.49 cm, respectively. The length of stalk in the GC and GP groups, were 21.63 ± 0.59 and 17.85 ± 0.16 cm, respectively. And the length of pepper stalk in CK group was 16.87 ± 0.22 cm. The length of pepper stalk in other four treatment groups were higher significantly than CK group while there was no significant difference between these treatment groups (Student t-test, p < 0.01). The POD activitiy were from 162.85 to 252.05 u/g and SOD activities were from 17.67 to 32.65 u/g, respectively. The activities of POD and SOD in BC, BP, GP group was significantly higher than that in CK group. Compared to the two groups treated with PSB06, there was no significant difference between BP group and GP group (Student t-test, p < 0.05). Compared with the two groups treated with *R. palustris* CGA009, BC group was significantly higher than GC group (Student t-test, p < 0.05).

**Fig. 2:**
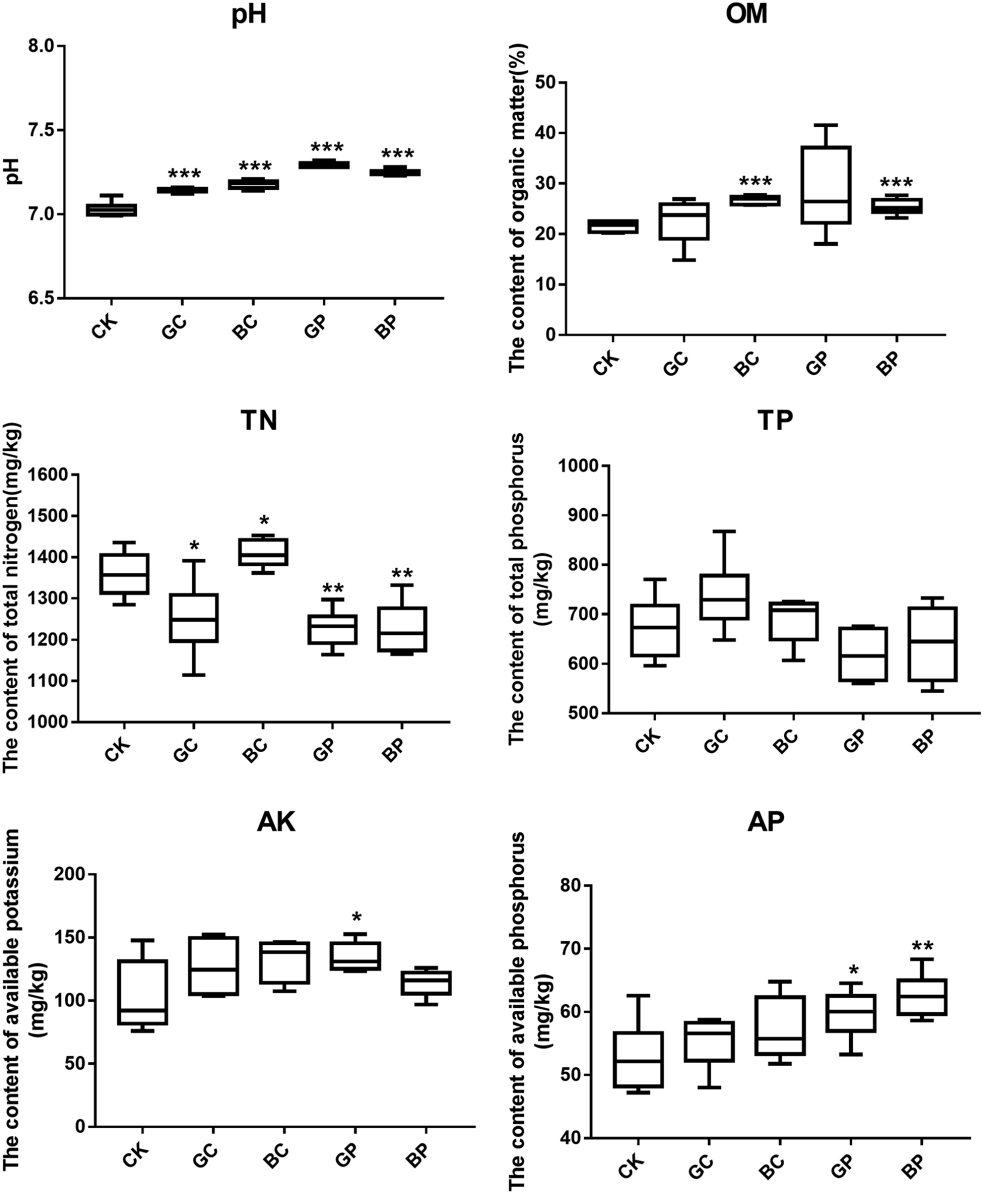
Physicochemical properties of rice soils. OM: organic matter, TN: total N, TP: total P, AK: available K, AP: available P. Control (CK): sterile water; treatment BP: the photosynthetic bacteria agent PSB06; treatment BC: the photosynthetic bacteria agent CGA009; treatment GP: root irrigation with the photosynthetic bacteria agent PSB06; treatment GC: root irrigation with the photosynthetic bacteria agent CGA009

The physicochemical properties of the 30 samples are summarized in Fig. 2 and Additional file 1: Table S1. Soil pH varied from 6.99 to 7.32. The Soil pH in other four treatment groups was higher significantly than the CK group (Student t-test, p < 0.001). And there was a significant difference between BC and BP, GC and GP, BC and GC, BP and GP group. The soil TN varied from 1114.16 to 1452.99 mg/kg, and other four treatment groups were higher significantly than the CK group (Student t-test, p < 0.01). The soil TP varied from 545.41 to 867.53 mg/kg among these soil samples, respectively. AK and AP varied from 75.93 to 152.6 and 47.21 to 68.34 mg/kg, respectively. The OM content among three groups varied from 14.86 to 41.58 mg/kg, BC and BP group was higher significantly than CK group.

### Response of bacterial communities among three groups

A total of 2,182,639 high-quality 16S rRNA gene sequences were obtained from 18 samples by Hiseq sequencing. Then 15,132 OTUs were generated after resampling with the minimum sequences. The dominant phyla and class were visualized in the Additional file 2: Figure S1 and the top 30 genera showed in the Additional file 2: Figure S2. In brief, the dominant phyla were *Proteobacteria* (51.95–61.66%), *Bacteroidetes* (5.40–9.39%), *Acidobacteria* (4.50–10.52%), *Actinobacteria* (5.06–8.14%), *Planctomycetes* (2.90–4.48%), *Chloroflexi* (2.23–5.06%) and *Firmicutes* (2.38–7.30%), accounted for 87% bacterial sequences. *Proteobacteria* and *Bacteroidetes* were the second most dominant phyla in the CK, BC and GP group while *Proteobacteria* and *Acidobacteria* in the BP and GC group. In the class level, the relative abundance of *Gammaproteobacteria* (35.62–44.88%), *Alphaproteobacteria* (7.35–12.73%), *Bacteroidia* (5.32–9.26%), *Deltaproteobacteria* (4.01–5.26%), *Subgroup 6* (2.30–5.85%) and *Actinobacteria* (2.92–4.41%) were observed in the three group with higher relative abundance (Additional file 2: Figure S2).

**Fig. 3:**
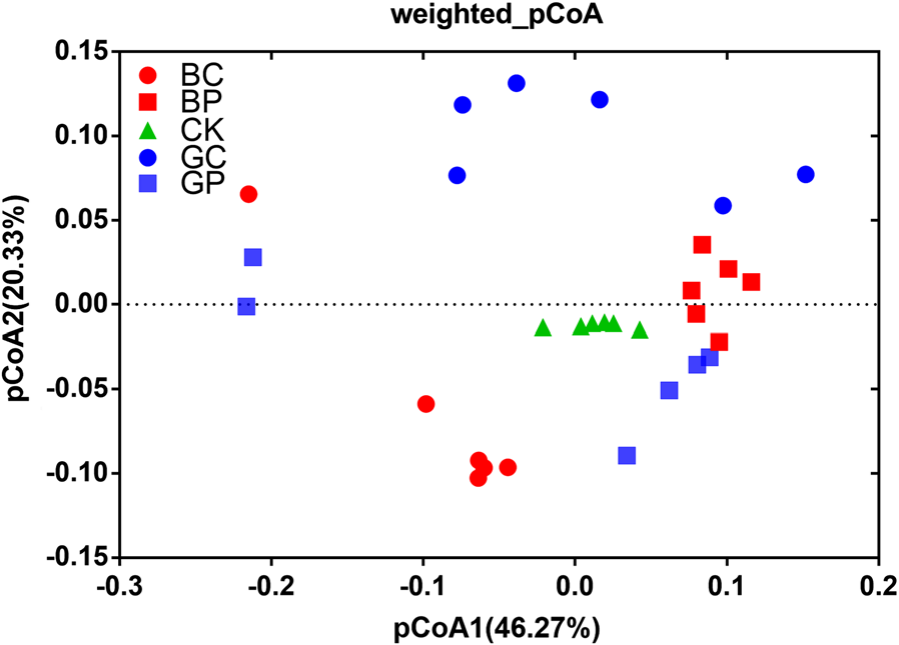
Principal coordinate analysis (weighted_PCoA) of microbial communities based on Bray–Curtis dissimilarity matrices. Control (CK): sterile water; treatment BP: the photosynthetic bacteria agent PSB06; treatment BC: the photosynthetic bacteria agent CGA009; treatment GP: root irrigation with the photosynthetic bacteria agent PSB06; treatment GC: root irrigation with the photosynthetic bacteria agent CGA009

The significant difference of alpha diversity between two groups was showed in the Table 1. The Chao1 value in GC group was lower significantly than CK group (Student t-test, p < 0.05), and there was no significant difference in Chao1 among other four groups (Student t-test, p > 0.05). Shannon index, In_simpson index, and Observerd_richness was no significant difference between four treatment groups and CK group (ANOVA, p > 0.05). The sample of three groups exhibited clear differences by pCoA. The pCoA result showed that CK group samples were clearly separated from other four group samples (Fig. 3). The first coordinate (pCoA1), showed 46.27% difference in bacterial population variation, and pCoA2 explained 20.33% dissimilarity (Fig. 3). In addition, it was further verified by the dissimilarity tests (MRPP, ANOSIM, and PERANOVA) based on Bray–Curtis distance and Jaccard distance among five groups (Additional file 1: Table S2, p < 0.05).

**Table 1:** Summary of α diversity index among different treatments

Group	Chao1	Shannon	Inv_Simpson	Observed_richness
CK	6297.08 ± 185.77a	6.5 ± 0.05ab	182.12 ± 5.81a	2579.17 ± 49.18a
BC	5818.56 ± 268.11a	6.06 ± 0.15b	100.67 ± 12b	2193.67 ± 115.51b
BP	6522.76 ± 78.01a	6.58 ± 0.03a	134.13 ± 10.79ab	2645.17 ± 20.97a
GC	4393.84 ± 318.11b	6.33 ± 0.14ab	149 ± 26.98ab	2399.17 ± 93.14ab
GP	5438.62 ± 653.15a	6.29 ± 0.24ab	151.07 ± 27.9ab	2386.83 ± 184.18ab

Control (CK): sterile water; treatment BP: the photosynthetic bacteria agent PSB06; treatment BC: the photosynthetic bacteria agent CGA009; treatment GP: root irrigation with the photosynthetic bacteria agent PSB06; treatment GC: root irrigation with the photosynthetic bacteria agent CGA009

Different letters indicate the statistical differences at a p value of < 0.05 for a one-way ANOVA

### The taxonomic abundance of dominant genera

The difference genera among the top 30 genera between two groups were assessed and showed in the Additional file 2: Figures S3, S4, S5. Among these genera, the relative abundance of *Klebsiella, Methyloversatilis, Anaerosinus Delftia, Massilia, Flavobacterium, Pseudomonas, Phenylobacterium, Dechloromonas and Rhodanobacter* have a significant difference between treatment groups and CK group. The relative abundance of *Massilia* in the GP group and *Pseudomonas, Rhodanobacter* in BP group was significantly lower than CK group. The relative abundance of *Pseudomonas, Flavobacterium* and *Klebsiella* in BC group was significantly higher than BP group. There was no significant difference in relative abundance between GC and GP groups. The relative abundance of *Rhodanobacter*, *Lysobacter* and *Massilia* in GP group was significantly lower than BP group, while *Flavobacterium* was significantly higher than BP group. The relative abundance of *Pseudomonas, Massilia, Flavobacterium* and *Klebsiella* in BC group was significantly higher than GC group.

### Relationships among dominant phyla, physicochemical properties and bacterial communities

**Fig. 4:**
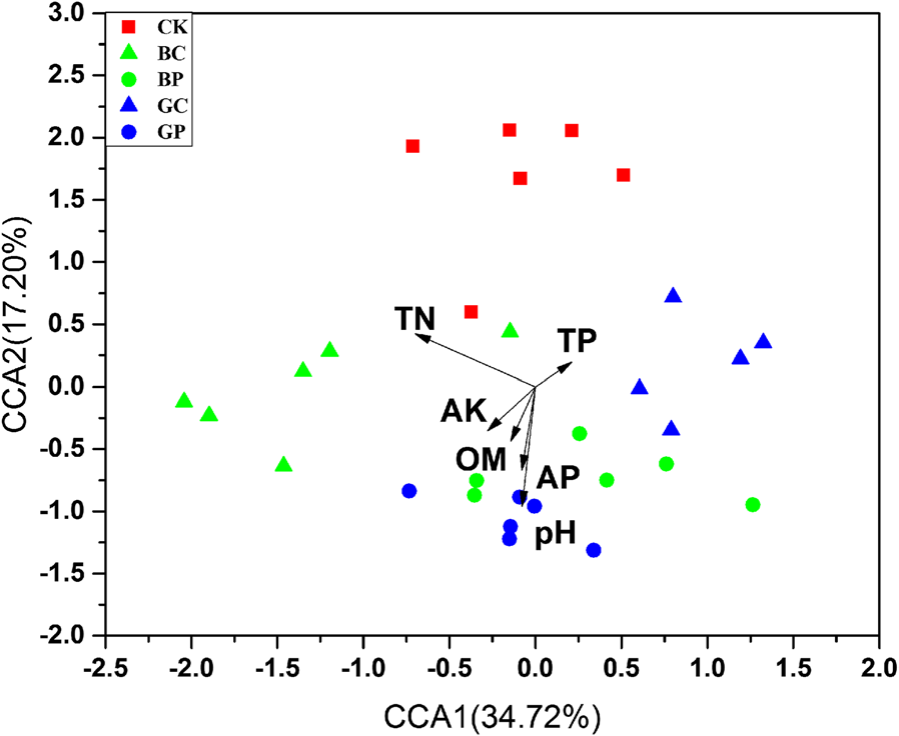
Canonical correspondence analyses (CCA) of the relative abundances of microbial communities with soil variables. OM: organic matter, TN: total N, TP: total P, AK: available K, AP: available P. Control (CK): sterile water; treatment BP: the photosynthetic bacteria agent PSB06; treatment BC: the photosynthetic bacteria agent CGA009; treatment GP: root irrigation with the photosynthetic bacteria agent PSB06; treatment GC: root irrigation with the photosynthetic bacteria agent CGA009

Canonical correspondence analysis (CCA) was used to investigate the relationships between dominant phyla, physicochemical properties and bacterial communities. Three clusters of bacterial communities were differentiated as the CCA result indicated (Fig. 4). CCA1 and CCA2 accounted for 34.72% and 17.20% of the total variation, respectively (Fig. 4). There were also significant positive correlations between bacterial community composition and the TN content (mantel test: p < 0.05, Additional file 1: Table S3). The relative abundance of dominant bacterial taxonomic groups varied among five groups. Significant negative correlation was observed between the TN and the relative abundance of *Acidobacteria* and *Thaumarchaeota* (p < 0.01), while the opposite trend was observed for the phylum *Proteobacteria* (r = 0.393, p < 0.05) (Additional file 1: Table S4). The pH was negative correlated with the relative abundance of *Gemmatimonadetes* (r = − 0.381, p < 0.05).

#### FAPROTAX analysis

All OTUs loaded 90 groups comprising 7820 members and established 2701 assignments of records to groups. There were 7675 out of 8970 records could not be assigned to any group, other records have been assigned to 54 groups. There were 49 same group in five groups. Besides, there was a significant difference between the four treatment groups and CK group in the major functional group types. And eight major functional groups were observed in all five groups; Compared with CK group, nitrogen_fixation, human_gut, and mammal_gut in the BC group, aerobic_ammonia_oxidation in the GC group, nitrogen_fixationhuman_gut and mammal_gut in the GP group was only observed as the main functional group (Additional file 2: Figure S6).

## Discussion

In this study, we provide new insight into the structure and composition of the root-associated microbiomes as well as showed the effect of the photosynthetic bacteria agent *R. palustris* PSB06 and CGA009 at the rice seedling stage with different treatment of seeds or seedlings. The previous study indicated that habitat, climate and geographical location, can affect the growth of crop and the formation of rhizosphere microorganisms (Wang et al. 2015; Zhang et al. 2015; Yang et al. 2016). For eliminate the interference of other factors, we conducted a greenhouse experiment, with day and night (16 h/8 h), to keep culture conditions at 28 °C. Sequencing of 16S rRNA gene amplicons provided especially a deeper look into the root-associated microbiomes of the rice at seedling stage, we were able to assess the variation of the microbial composition by different treatments. In our study, we showed that PSB06 have not only a significant impact on plant growth, but also on the structure and composition of the root-associated microbiome. Here, the PSB06 can significantly improve stress resistance of rice at seedling stage. However, for bacterial agent *R. palustris* CGA009, only the stress resistance has been enhanced with seed soaking treatment.

Soil microorganism is a key component of soil ecological environment. And the diversity of the soil microorganism is an important index to evaluate the status of microecological environment (Manching et al. 2014). Previous studies indicated that we can control plant disease incidence by increasing the soil bacterial diversity and regulating some bacterial abundances (Van Elsas et al. 2012; Yang et al. 2017; Xiao et al. 2018). There was a remarkable difference in their overall composition, their dominant populations, and diversity indices among five treatment group in this study. Here, our study showed that α diversity index (Shannon index, Inv_Simpson index and Observed_richness) has no significant difference between the CK group and other groups that treated with *R. palustris* PSB06 agents (Table 1). At same time, significant differences in bacterial community composition were observed between two treatments based on the dissimilarity test and pCoA results (Additional file 1: Table S1, Fig. 3). The dissimilarity test and pCoA results also indicated that there was a significant difference with different treatment methods by same agent.

The application of biocontrol agents for plant pathogens is a promising solution for sustainable agriculture. Until today, the mechanisms described for biocontrol agents focus on direct antagonistic effects against a pathogen or an interaction via the plant’s immune system (Doornbos et al. 2012). While *R. palustris* PSB06 belongs to photosynthetic bacteria, which has been studied as an efficient biocontrol agent. And the *R. palustris* PSB06 also has good promotion effect on the crop (Zeng et al. 2018). In this study, the application patterns of seed immersion and root irrigation had similar effects on rice, both of which could effectively promote the growth of stem. Meanwhile, the POD and SOD activity in *R. palustris* PSB06 treatment group were higher than the control group, indicated that the *R. palustris* PSB06 also can effectively enhance the stress resistance of rice at seedling stage. Plant–microbe interactions usually lead to changes in the abundance of some beneficial bacteria or pathogenic bacteria strains, and then may result in nonlinear changes in the composition of the entire microbiome. It may have a negative impact on plants and humans (Berg et al. 2005). To compare the effects of different agricultural application patterns on rhizosphere microorganisms at seedling stage, we analyzed the population among different groups at the genus level. The genus *Rhodanobacter* dominate bacterial communities were often observed in the highly contaminated environment (Green et al. 2012; Carlson et al. 2019). Pseudomonas is a model organism to study beneficial plant–microbe interactions (Haas and Défago 2005), which related to healthy or diseased plants (Adesina et al. 2009). In our study, the relative abundance of *Rhodanobacter*, *Lysobacter* and *Massilia* in GP group was significantly lower than BP group, while *Flavobacterium* was significantly higher than BP group. While the relative abundance of *Pseudomonas, Massilia, Flavobacterium* and *Klebsiella* in BC group was significantly higher than GC group. FAPROTAX afford a simple functional prediction method for Prokaryotic Taxa (Louca et al. 2016). In our study, the results indicated there was a significant difference between the four treatment groups and CK group in the major functional group types. FAPROTAX defines functional groups in terms of taxa (e.g. species or genera) affiliated with each functional group. These affiliations are mostly based on peer-reviewed literature, such as announcements of cultured representatives. For species and functional analysis, metagenomic and transcriptomic techniques are required in the future study.

The physicochemical properties of soil were considered to be one of the important factors to change plant rhizosphere microorganisms in previous studies (Zhou et al. 2017). Although the soil physicochemical properties were similar when seeds were first planted, the surface environment of rice seedlings may change greatly after a long period of growth treated with the *R. palustris* PSB treatment, and then affect the rhizosphere environment. In this study, the relationships between physicochemical properties, dominant populations and microbial communities were discussed. The mantel test and CCA results showed that pH, TN and OM were significantly correlated with microbial communities (Additional file 1: Table S3, Fig. 4). The soil pH value has been considered as an important factor affecting plant rhizosphere microorganisms, and plays an important role in changing microbial community diversity and structure (Zhou et al. 2017). The results showed that the pH of the treatment group was significantly higher than the CK group (Fig. 2, Additional file 1: Table S1), indicating that the treated group consumed less soil organic matter or the crops produced more organic matter. The OM of the seeding group were higher than the CK group, and the PSB treatment group that soaking with seeds were higher significantly than the CK group (Fig. 2, Additional file 1: Table S1). This result indicated that the metabolism of the treated group was enhanced, which may be that the growth and development of crops were promoted after the treatment. There was a significant difference in total nitrogen between the treatment group and CK group. Interestingly, the total nitrogen content of the GC, GP and BP group was lower than the CK group, while the total nitrogen content of BC group was significantly higher than the CK group. There was a significant difference in total nitrogen between two treatments of the *R. palustris* CGA009. And there was also a significant difference between two treatments of the *R. palustris* PSB06 and CGA009, which may be caused by the different nutrition modes of the two bacteria. The *R. palustris* PSB06 can effectively reproduce under anaerobic conditions, while *R. palustris* CGA009 is more suitable for aerobic environment. We also analyzed the correlation between physical and chemical properties and the dominant population, and the results showed that TN, AK and OM were significantly correlated with the dominant population. The TN was negatively correlated with the relative abundance of *Acidobacteria* and positively correlated with the phylum *Proteobacteria* (Additional file 1: Table S4). The pH was negatively correlated with the relative abundance of *Gemmatimonadetes* (r = − 0.381, p < 0.05).

## Supplementary information

**Additional file 1.** Additional Tables.

**Additional file 2.** Additional Figures.

## Data Availability

All data obtained have been included into the manuscript and its additional files.

## Abbreviations

POD: peroxidase
SOD: superoxide dismutase
CCA: canonical correspondence analysis
BCAs: biological control agents
pCoA: the principal coordinate analysis
MRPP: nonparametric multi-response permutation procedures
ANOSIM: analysis of similarities
ADONIS: non-parametric permutational multivariate analysis of variance of the Adonis function
