# Supplementary material for: The effects of Rhodopseudomonas palustris PSB06 and CGA009 with different agricultural applications on rice growth and rhizosphere bacterial communities
Source: AMB Express. 2019 Oct 31;9:173. doi: 10.1186/s13568-019-0897-z (PMC6823419; doi:10.1186/s13568-019-0897-z)
Supplement: Supplementary file 1 — Additional file 1. Additional Tables. [file 13568_2019_897_MOESM1_ESM.docx]

**Table S1 The dissimilarity test of the psychemical porties between two group based on the stdent-T-test.** * indicates that the signiﬁcant value p < 0.05, ** indicates that the signiﬁcant value p < 0.01, *** indicates that the signiﬁcant value p < 0.001. Control (CK): sterile water; treatment BP: the photosynthetic bacteria agent PSB06; treatment BC: the photosynthetic bacteria agent CGA009; treatment GP: root irrigation with the photosynthetic bacteria agent PSB06; treatment GC: root irrigation with the photosynthetic bacteria agent CGA009.

| **Groups** | **pH** | **AK** | **TN** | **TP** | **AP** | **OM** |
| --- | --- | --- | --- | --- | --- | --- |
| **CK-BC** | *** | 0.0512 | * | 0.6310 | 0.1895 | *** |
| **CK-BP** | *** | 0.3779 | ** | 0.4451 | ** | *** |
| **CK-GC** | *** | 0.1437 | * | 0.1265 | 0.4151 | 0.5996 |
| **CK-GP** | *** | * | ** | 0.1283 | * | 0.0721 |
| **BC-BP** | *** | * | *** | 0.2140 | * | 0.0927 |
| **GC-GP** | *** | 0.4789 | 0.5918 | ** | 0.0846 | 0.1523 |
| **BC-GC** | ** | 0.6544 | ** | 0.1880 | 0.4799 | * |
| **BP-GP** | *** | ** | 0.9582 | 0.5383 | 0.1881 | 0.3877 |

**Table S2** **Dissimilarity test (MRPP, ANOSIM and ADONIS) of bacterial communities between two different groups.** Control (CK): sterile water; treatment BP: the photosynthetic bacteria agent PSB06; treatment BC: the photosynthetic bacteria agent CGA009; treatment GP: root irrigation with the photosynthetic bacteria agent PSB06; treatment GC: root irrigation with the photosynthetic bacteria agent CGA009.

|  | **MRPP** | | | | **ANOSIM** | | | | **ADONIS** | | | |
| --- | --- | --- | --- | --- | --- | --- | --- | --- | --- | --- | --- | --- |
|  | **Bray-Curtis** | | **Jaccard** | | **Bray-Curtis** | | **Jaccard** | | **Bray-Curtis** | | **Jaccard** | |
|  | **δ** | **P** | **δ** | **P** | **R** | **P** | **R** | **P** | **F** | **P** | **F** | **P** |
| **CK-BC**  **CK-BP**  **CK-GC** | 0.2651 | 0.003 | 0.5415 | 0.006 | 0.9055 | 0.002 | 0.7074 | 0.002 | 5.5905 | 0.002 | 1.5424 | 0.004 |
|  | 0.2575 | 0.002 | 0.5254 | 0.007 | 0.9731 | 0.002 | 0.5629 | 0.002 | 5.8246 | 0.002 | 1.2562 | 0.003 |
|  | 0.3119 | 0.004 | 0.5409 | 0.002 | 0.624 | 0.004 | 0.4666 | 0.002 | 4.2418 | 0.003 | 1.4012 | 0.002 |
| **CK-GP** | 0.3243 | 0.003 | 0.557 | 0.223 | 0.45 | 0.006 | 0.1314 | 0.069 | 2.3029 | 0.019 | 1.0786 | 0.235 |
| **BC-BP** | 0.2583 | 0.004 | 0.5361 | 0.006 | 0.9962 | 0.002 | 0.7129 | 0.004 | 9.0181 | 0.003 | 1.7398 | 0.003 |
| **GC-GP** | 0.372 | 0.026 | 0.5671 | 0.021 | 0.2648 | 0.051 | 0.2537 | 0.021 | 2.3898 | 0.028 | 1.3175 | 0.021 |
| **BC-GC** | 0.3128 | 0.006 | 0.5516 | 0.001 | 0.887 | 0.001 | 0.8796 | 0.003 | 7.2726 | 0.003 | 1.9797 | 0.003 |
| **BP-GP** | 0.3176 | 0.002 | 0.5517 | 0.005 | 0.6277 | 0.002 | 0.4481 | 0.001 | 3.9306 | 0.005 | 1.366 | 0.005 |

**Table S3 The mantel test results between environmantal facters and bacterial communities based on Bray-Curtis and Jaccard distances.** r.BC and p.BC: the r and p value for Bray-Curtis distance; r.JC and p.JC: the r and p value for Jaccard distance.

| **Envs** | **r.BC** | **p.BC** | **r.JC** | **p.JC** |
| --- | --- | --- | --- | --- |
| **pH** | -0.0104 | 0.504 | -0.0944 | 0.816 |
| **AK** | -0.0814 | 0.857 | -0.1299 | 0.929 |
| **TN** | 0.2210 | 0.006 | 0.2113 | 0.012 |
| **TP** | 0.1000 | 0.162 | 0.1093 | 0.156 |
| **AP** | -0.0552 | 0.729 | -0.1057 | 0.841 |
| **OM** | 0.2716 | 0.033 | 0.2766 | 0.059 |

**Table S4 Spearman correlation(r) coefficients between soil properties and dominant bacterial populations.** * indicates that the signiﬁcant value P < 0.05, ** indicates that the signiﬁcant value P < 0.01. OM: organic matter, TN: total N, TP: total P, AK: available K, AP: available P.

| **r** | | **pH** | **AK** | **TN** | **TP** | **AP** | **OM** |
| --- | --- | --- | --- | --- | --- | --- | --- |
| ***Proteobacteria*** | -0.148 | | 0.129 | 0.393* | 0.181 | 0.0452 | -0.00467 |
| ***Bacteroidetes*** | 0.151 | | 0.151 | 0.244 | -0.252 | -0.0283 | 0.156 |
| ***Acidobacteria*** | 0.0288 | | -0.249 | -0.481** | 0.0354 | 0.0363 | -0.218 |
| ***Actinobacteria*** | -0.0018 | | -0.0647 | -0.0861 | -0.0207 | -0.198 | -0.0492 |
| ***Firmicutes*** | 0.246 | | 0.291 | 0.03 | -0.129 | 0.157 | 0.205 |
| ***Chloroflexi*** | -0.17 | | -0.167 | -0.27 | 0.0768 | -0.221 | -0.287 |
| ***Planctomycetes*** | 0.044 | | -0.17 | -0.129 | -0.128 | -0.0616 | -0.13 |
| ***Gemmatimonadetes*** | -0.381* | | -0.319 | 0.195 | -0.103 | -0.099 | -0.246 |
| ***Verrucomicrobia*** | -0.177 | | -0.308 | 0.202 | -0.0603 | 0.104 | -0.0176 |
| ***Thaumarchaeota*** | -0.0437 | | -0.114 | -0.516** | 0.0229 | 0.0225 | -0.215 |
| ***Rokubacteria*** | -0.0078 | | -0.0596 | -0.239 | 0.0418 | -0.258 | -0.202 |
| ***Cyanobacteria*** | -0.0607 | | 0.0624 | -0.199 | -0.144 | 0.22 | 0.0223 |
