# Supplementary material for: The effects of Rhodopseudomonas palustris PSB06 and CGA009 with different agricultural applications on rice growth and rhizosphere bacterial communities
Source: AMB Express. 2019 Oct 31;9:173. doi: 10.1186/s13568-019-0897-z (PMC6823419; doi:10.1186/s13568-019-0897-z)
Supplement: Supplementary file 2 — Additional file 2. Additional Figures. [file 13568_2019_897_MOESM2_ESM.docx]

**
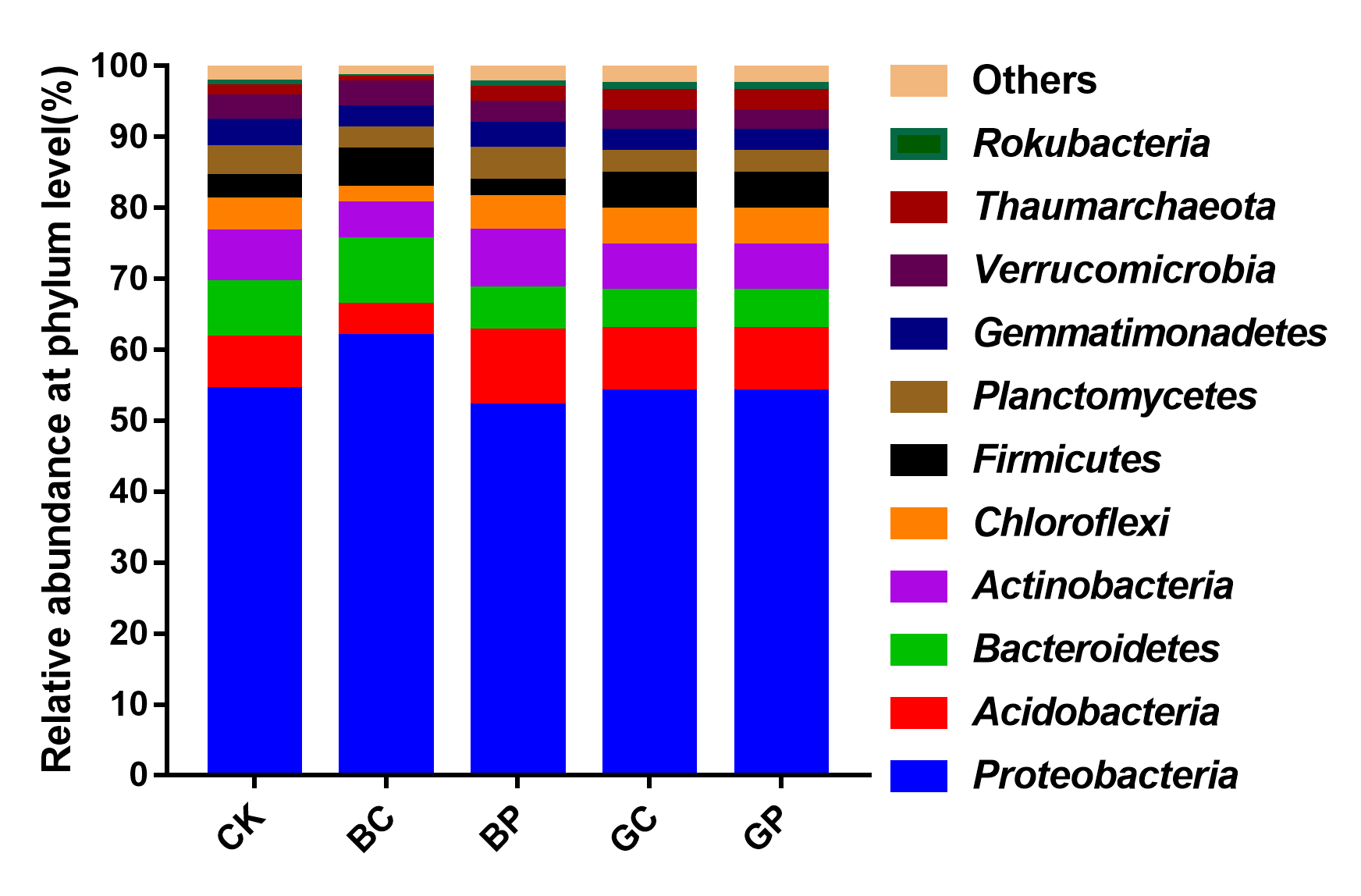
**

**Figure S1 Relative abundance of bacterial classification at the phylum level.** Control (CK): sterile water; treatment BP: the photosynthetic bacteria agent PSB06; treatment BC: the photosynthetic bacteria agent CGA009; treatment GP: root irrigation with the photosynthetic bacteria agent PSB06; treatment GC: root irrigation with the photosynthetic bacteria agent CGA009.


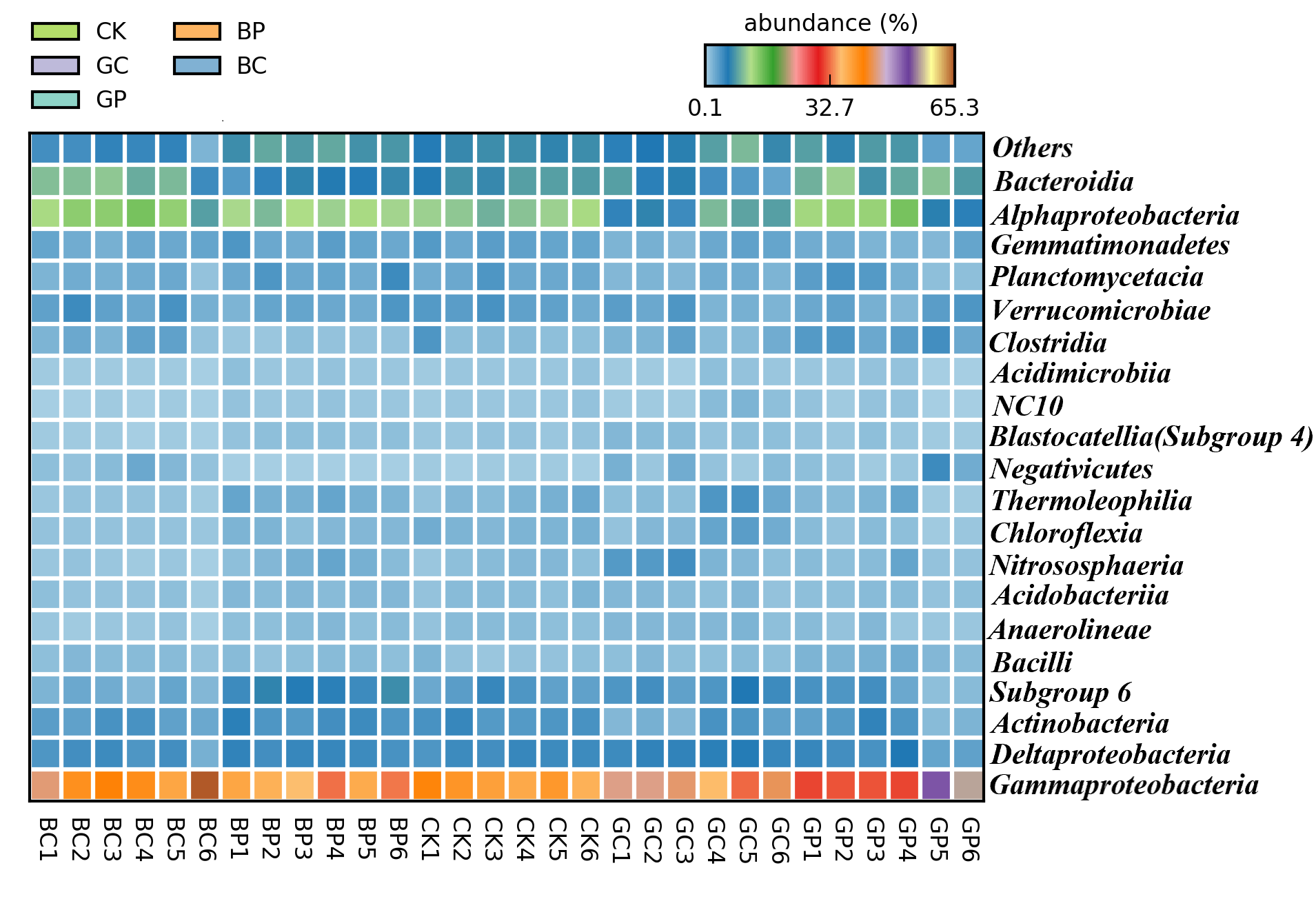


**Figure S2 Relative abundance of bacterial classification at the class level.** Control (CK): sterile water; treatment BP: the photosynthetic bacteria agent PSB06; treatment BC: the photosynthetic bacteria agent CGA009; treatment GP: root irrigation with the photosynthetic bacteria agent PSB06; treatment GC: root irrigation with the photosynthetic bacteria agent CGA009.

**
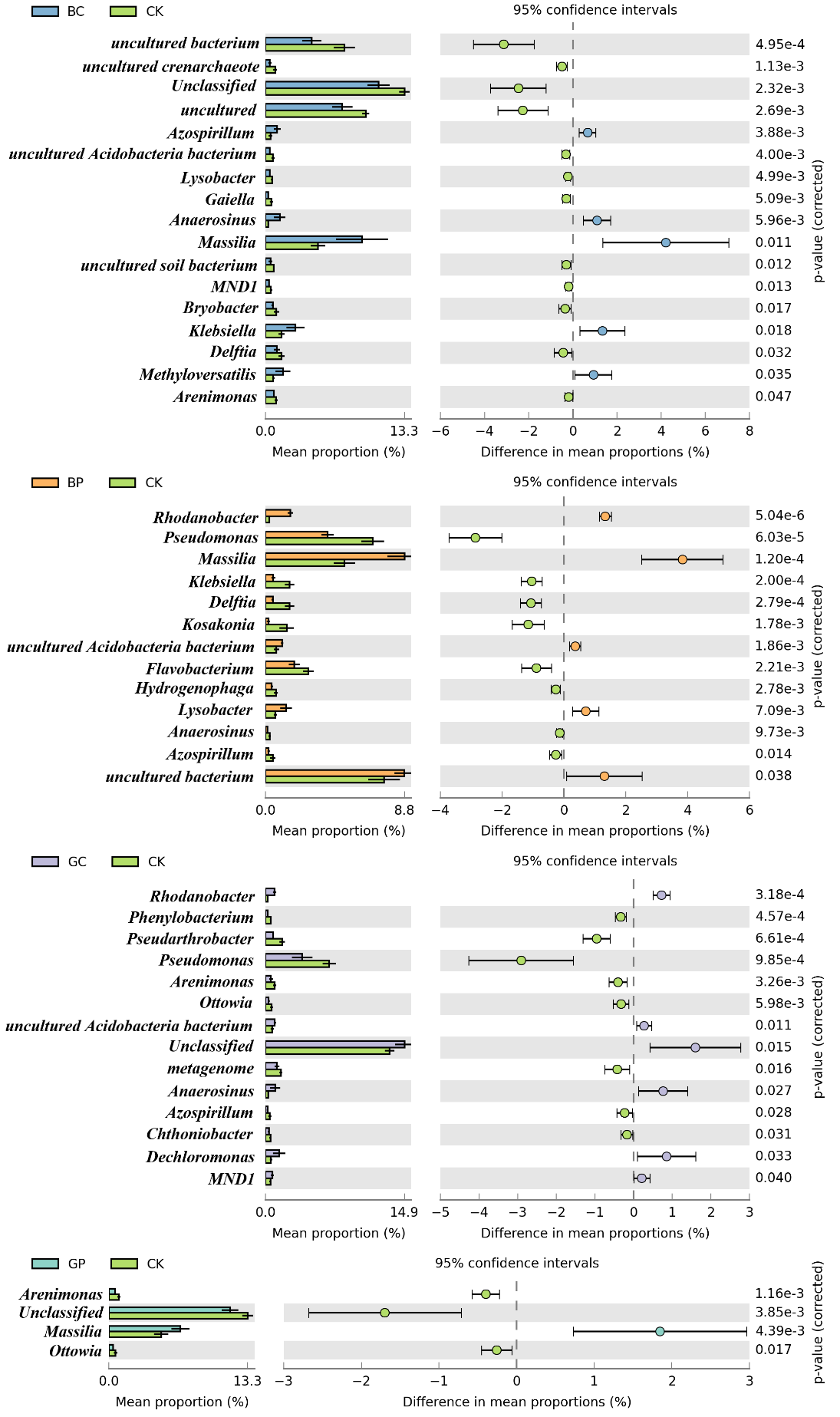
**

**Figure S3 The dominant genus differences between CK and other treatments (BC, BP, GC and GP).** Control (CK): sterile water; treatment BP: the photosynthetic bacteria agent PSB06; treatment BC: the photosynthetic bacteria agent CGA009; treatment GP: root irrigation with the photosynthetic bacteria agent PSB06; treatment GC: root irrigation with the photosynthetic bacteria agent CGA009.

**
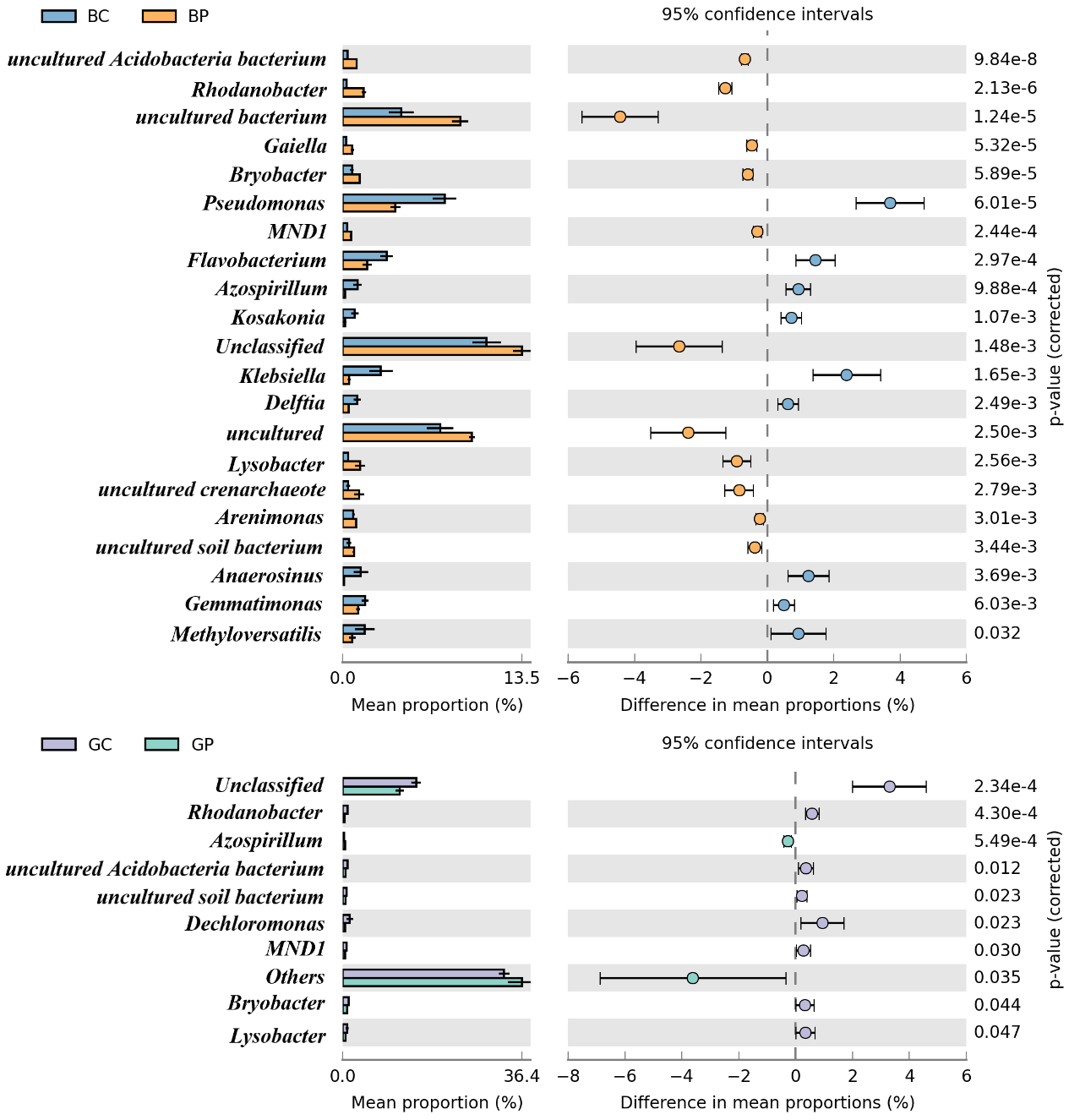
**

**Figure S4 The** **dominant genus differences at two different agents with same agricultural application.** Control (CK): sterile water; treatment BP: the photosynthetic bacteria agent PSB06; treatment BC: the photosynthetic bacteria agent CGA009; treatment GP: root irrigation with the photosynthetic bacteria agent PSB06; treatment GC: root irrigation with the photosynthetic bacteria agent CGA009.

**
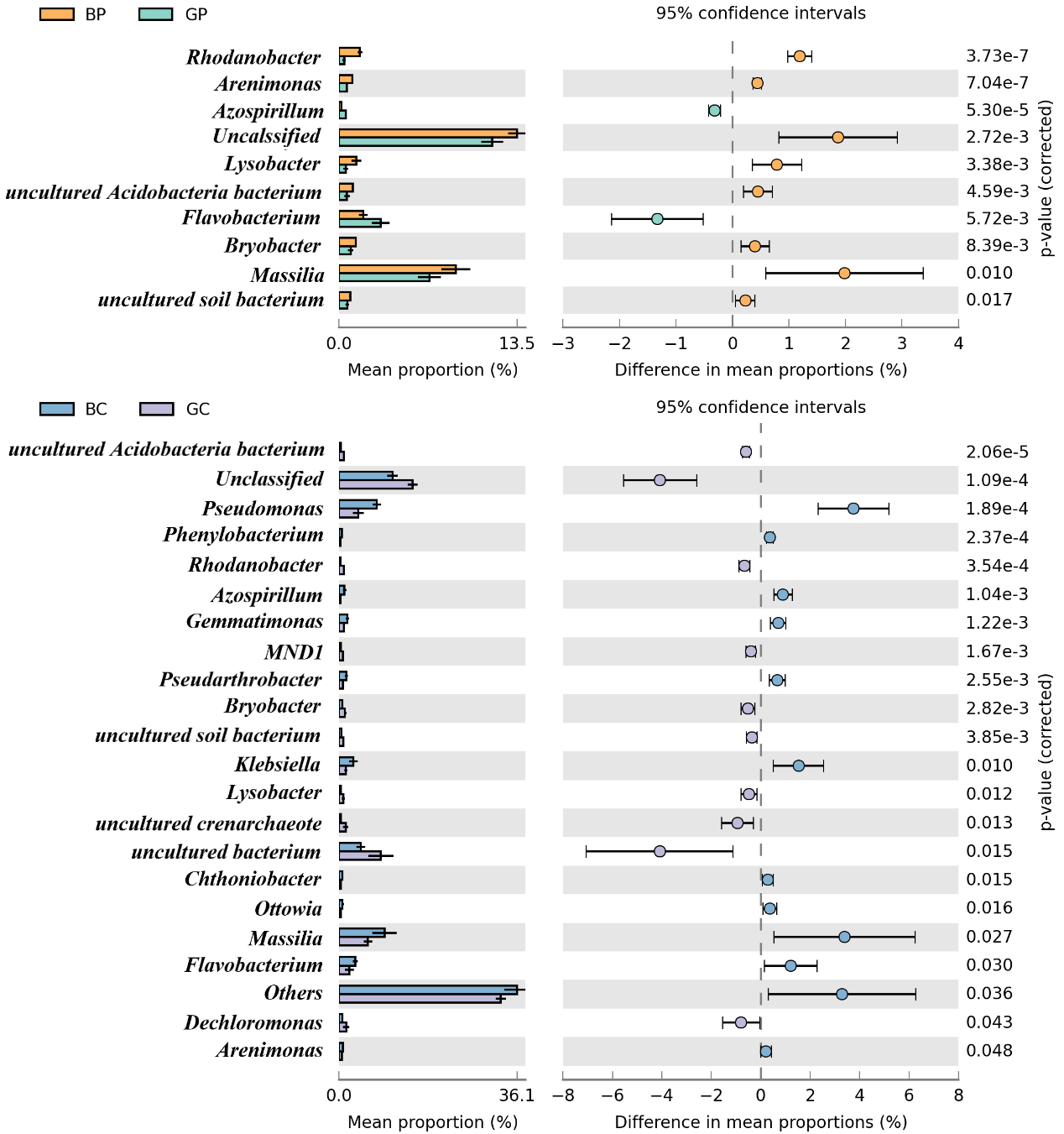
**

**Figure S5 The dominant genus differences at same agents with different agricultural application.** Control (CK): sterile water; treatment BP: the photosynthetic bacteria agent PSB06; treatment BC: the photosynthetic bacteria agent CGA009; treatment GP: root irrigation with the photosynthetic bacteria agent PSB06; treatment GC: root irrigation with the photosynthetic bacteria agent CGA009.

**
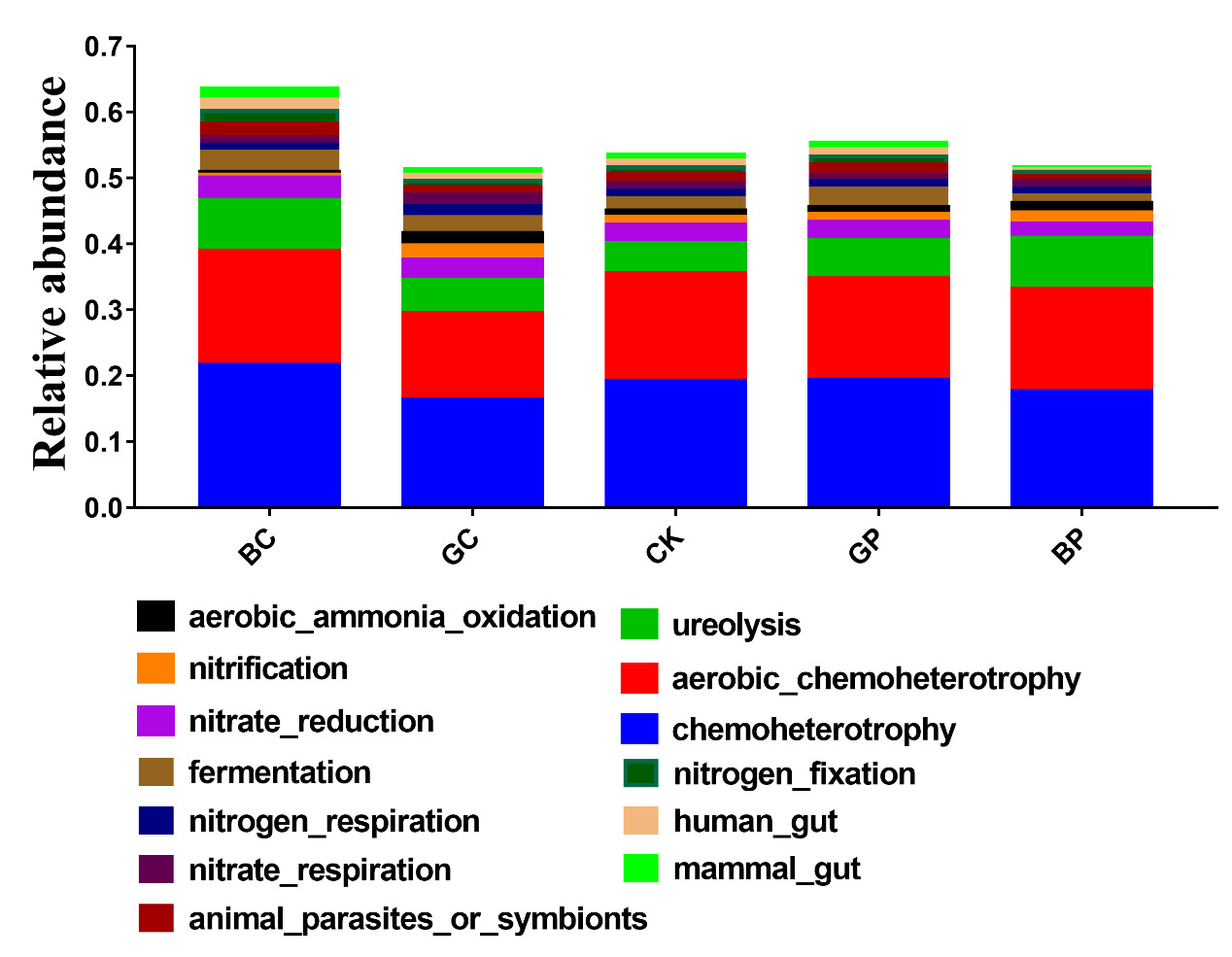
**

**Figure S6 The dominant functional groups at same agents with different agricultural application.** Control (CK): sterile water; treatment BP: the photosynthetic bacteria agent PSB06; treatment BC: the photosynthetic bacteria agent CGA009; treatment GP: root irrigation with the photosynthetic bacteria agent PSB06; treatment GC: root irrigation with the photosynthetic bacteria agent CGA009.
